# 

**Published:** 1833-08

**Authors:** 


### MONTHLY LIST OF MEDICAL BOOKS.

[*Medical Works cannot be entered on this List unless a copy be sent for the purpose,—the titles of Books having frequently been sent to us as published, which have not appeared for weeks, or even months, after.*]

The Teeth, in relation to Beauty, Voice, and Health; being the Result of Twenty Years' practical Experience and assiduous Study to produce the full Development and perfect Regularity of those essential Organs. By JOHN NICHOLLS, Surgeon-Dentist.—Large 8vo. pp. 134. Hamilton and Co., London.

An Essay on Inflammation; being an Inquiry into the Causes, Phenomena, Treatment, and Terminations of this Condition, with a View to the Elucidation of the Proximate Cause. By PHILIP LOVELL PHILLIPS, M.D. Oxon.—8vo. pp. 153. Burgess and Hill, London.

Part XXI. The Principles and Practice of Obstetric Medicine, in a Series of Systematic Dissertations on Midwifery and on the Diseases of Women and Children. Illustrated by numerous Plates. By D. D. DAVIS, M.D. M.R.S.L., &c.—4to. John Taylor, London.

Letters on the Cholera Asphyxia, as it has appeared in the City of New York: addressed to JOHN C. WARREN, M.D., of Boston, and originally published in that city. Together with other Letters, not before published. By MARTYN PAINE, M.D.—8vo. pp. 160. Collins and Hanway, New York.

A Treatise on the Composition and Medical Properties of the Mineral Waters of Buxton, Matlock, Tunbridge Wells, Harrogate, Bath, Bristol, Cheltenham, Leamington, Malvern, Isle of Wight, Brighton, and the Beulah Spa, Norwood. With instructive Observations on the Drinking of the Waters, and the use of the several Baths. By Sir CHARLES SCUDAMORE, M.D. F.R.S. &c. Second Edition, corrected and much enlarged.—8vo. pp. 215. Longman and Co., London.

### APOTHECARIES' HALL.

Names of Gentlemen to each of whom the Court of Examiners have granted Certificates:

JULY 4.

George Archer, Mildenhall
Nathaniel Collyer, Gislingham
John Say Clarke, Lincolnshire
Wm. Neyle Cornish, Devonshire
Luther Owen Fox, Broughton
John Gayleard
Samuel Price Prichard, Shrewsbury
Gerald O'Reilly.

JULY 11.

John Moore Bates
William Bainbridge, York
Maurice Dyte, London
Thomas Fentem, Eyam, Derbyshire
Hamilton Mason, Bishopwearmouth
Hildebrand Phipos, Hackney Road
William John Tubbs, Clenchwater
George Montague Scott Seaward, Clapham
Francis Robert Smith Upjohn, Field Dalling
Charles Wordingham, Norfolk.

JULY 18.

John Greeves, Aldersgate street
John Thomas Morris, Jersey
John James Ridge, Bridge Road, Lambeth
Richard Thomas Russell, Tonbridge, Kent
Wm. Samuel Slinn, Whittlesea, Camb.

JULY 25.

Thomas Brent, Bratton, Wilts.
Benjamin Dodsworth, Wheldrake, York.
Robert Gibbon, Wateringbury
Thomas Long, Portsmouth.

